# Orthoplastic surgery for interdisciplinary extremity reconstruction

**DOI:** 10.1515/iss-2025-0026

**Published:** 2025-11-04

**Authors:** Anja M. Boos, Heide Delbrück, Philipp Lichte, Benedikt Schäfer, Alexander Gombert, Christian Uhl, Frank Hildebrand, Justus P. Beier

**Affiliations:** Department of Plastic Surgery, Hand Surgery – Burn Center, University Hospital RWTH Aachen, Pauwelsstr. 30, 52074 Aachen, Germany; Department of Plastic, Reconstructive and Aesthetic Surgery – Hand Surgery and Burn Center, University Hospital Schleswig-Holstein, Campus Lübeck, Germany; Department of Orthopaedics, Trauma and Reconstructive Surgery, University Hospital RWTH Aachen, Aachen, Germany; Department of Vascular Surgery, University Hospital RWTH Aachen, Aachen, Germany

**Keywords:** orthoplastic surgery, interdisciplinarity reconstruction, extremity reconstruction, individualized therapy

## Abstract

**Objectives:**

Combined bone and soft tissue injuries and defects pose a challenge for the interdisciplinary orthoplastic surgical team. These conditions are not only encountered in young, healthy patients, but, due to various causes ranging from trauma over chronic osteomyelitis to tumor resection, also in patients with pre-existing vascular disease and even many years after the initial trauma.

**Methods:**

An efficient interdisciplinary treatment approach by orthopedic/trauma surgeons and plastic surgeons, and if necessary, vascular surgeons, who develop a joint strategy as early as possible is crucial for optimal functional restoration of form and function.

**Results:**

The goal of this orthoplastic approach is early definitive fracture stabilization or bony reconstruction combined with adequate soft tissue coverage and reconstruction of other injured structures such as vessels, nerves, and tendons, in order to begin rehabilitation as early as possible.

**Conclusions:**

The different steps of the orthoplastic surgical approach for interdisciplinary extremity reconstruction is described in the following manuscript and should encourage and provide a guideline for other orthoplastic teams, possibly leading to establishing specialized extremity reconstruction centers.

## Introduction

### Why orthoplastic surgery is needed for optimized extremity reconstruction – clinical problem and state of the art

Regardless of the etiology, lower extremity salvage and reconstruction attempts to avoid amputation, restore limb function, and improve quality of life outcomes. This goal requires a treatment team well trained in skeletal and soft tissue reconstruction, vascular and other systemic pathologies and finally physical rehabilitation [1]. Historically, orthopedic and plastic surgeons worked separately when faced with challenging reconstructive cases involving lower extremity skeletal and soft tissue reconstruction. With time, many embraced that their seemingly separate skill sets, and knowledge could be unified in a collaborative orthoplastic approach to offer patients the best possible chance for success. Beyond the concerted care of orthopedic and plastic surgeons, limb salvage today benefits from input from many other specialties including musculoskeletal radiologists, vascular surgeons, infectious disease specialists, physical therapists, prosthetists, and specialized nursing staff [1]. Orthoplastic surgery optimizes extremity reconstruction by combining the expertise of orthopedic and plastic surgeons to address both bony and soft tissue issues. This collaborative approach leads to faster bone union, more durable soft tissue coverage, and improved functional outcomes, ultimately minimizing the need for revision procedures and amputation [2]. The microsurgical reconstructive ladder ascends from basic microsurgical procedures such as a digital artery or nerve repair to more complex procedures such as autologous tissue transplantation. Functional muscle transfers, toe-to-hand transfers, and recently vascularized composite allotransplantation are the highest rungs on this ladder that help restore extremity function [3]. The implementation of protocols, systems, and centers that foster this approach leads to improved outcomes for these patients [4]. When faced with challenging cases of chronic osteomyelitis, nonhealing wounds in diabetic patients, large tumors, or high grade traumatic injuries, Azoury–Levin and colleagues encourage centers to embrace the orthoplastic approach when considering limb salvage, as the decision to amputate is irreversible [1]. The remarkable growth in orthoplastic surgery research over the past decade provides a strong foundation for improving patient care and expanding collaboration between plastic and orthopedic surgeons [2]. The goal of limb salvage is to have the patient achieve the best possible function that allows daily activities and the ability to perform tasks that require strength and dexterity. This is a complex process requiring the work of a multidisciplinary team practicing the principles of good wound care, orthoplastic thinking, and strategizing on future functions. Understanding the new advances in this field of rapid change and growth will help reconstructive surgeons to make better decisions and incorporate them into daily practice, allowing for the best possible outcome [5].

## Methods

### Interdisciplinary case planning to optimize patient care and early fracture stabilization

The large number of different injury patterns requires very variable approaches to the reconstruction of bone and soft tissue defects and other functional structures. This makes standardized treatment more difficult and requires tailor-made solutions, which ideally include interdisciplinary care with orthopedic/trauma surgeons, plastic surgeons, infectiologists and vascular surgeons as well as other necessary specialist disciplines, such as internal specialists and psychiatric/psychological support for patients with cardiac and/or renal disease. Once the therapy concept has been determined in an interdisciplinary manner, a coordinated, multi-step surgical procedure is usually carried out by the various surgical disciplines. Fracture-related infection is a serious post-traumatic complication that is occasionally accompanied by inadequate or even non-vital soft tissue cover. In these cases, a carefully planned orthoplastic approach is mandatory, as a vital and intact soft tissue envelope is essential to achieve fracture healing and infection healing. This subgroup of patients is complex and complication-prone, has a high risk of impaired fracture consolidation and recurrent infections, and is expected to have a long healing process even with optimal planning. A multi-step, multidisciplinary approach with close coordination of timing between the trauma surgery/orthopedic and plastic surgery team is increasingly being advocated and increases the quality of care [6], 7]. Especially for lower leg fractures, several studies demonstrate how important the coordination between definitive fracture care and soft tissue defect coverage is for optimal patient care [8], 9].

Close early joint case review and therapy planning is the key to optimizing patient care. This has already been successfully established in other countries. The “BOAST 4 guidelines – STANDARD for TRAUMA” defines the close interdisciplinary collaboration between the “British Orthopedic Association” and the “British Association of Plastic, Reconstructive and Aesthetic Surgeons”. Multidisciplinary management in specialized centers is an important basic requirement here. According to BOAST, fracture stabilization should take place in the first 24 h – defect coverage as orthoplastic surgery in the first 72 h. In the UK this results in reduced complication rates with fewer infections and limb loss. The “fix and flap” technique advocated for selected cases reduces the surgical burden on patients [10], [11], [12].

The “American Academy of Orthopedic Surgeons – AAOS” has also summarized recommendations for orthoplastic procedures in the “Clinical Practice Guideline Summary: Prevention of Surgical Site Infection After Major Extremity Trauma”. This guideline includes 14 recommendations for pre-, peri- and postoperative procedures to reduce wound infections after extensive extremity trauma and help to identify and evaluate patient-specific risk factors. The recommended implementation of an orthoplastic team potentially results in a reduction in deep wound infections, the number of bone procedures required, and shortens the time to wound healing and fracture consolidation. In addition, economic considerations can be addressed by streamlining the length of stay and return to work [13], [14], [15].

### Preoperative evaluation of the patient and the vascular condition

Preoperative evaluation for surgery involves a comprehensive assessment of the patient’s overall health and the specific vascular supply to the extremity to be operated on. This process helps identify potential risks, optimize the patient’s condition for surgery, and personalize the surgical approach. A thorough history, including cardiac and pulmonary symptoms, functional capacity, and risk factors for cardiac, pulmonary, and infectious complications, is crucial. This should include any known history of cardiac conditions, diabetes, hypertension, etc. Evaluate the patient’s ability to perform daily activities to determine their functional status and potential risks associated with the surgery. The type of surgery and the patient’s underlying health conditions influence the overall perioperative risk. High-risk patients, such as those with unstable angina or recent myocardial infarction, require more extensive evaluation. Assess the patient’s nutritional status, as malnutrition can increase the risk of complications [16], 17].

A history related to vascular disease, such as peripheral artery disease, must be thoroughly addressed prior to surgery. Evaluation of the involved vascular territory, including pulses, skin changes, and ulcerations is essential. Imaging techniques like angiography, ultrasound, or CT angiography may be used to visualize the vascular supply and identify any abnormalities or stenosis. The findings from the vascular assessment may lead to a revision of the planned surgical approach or vascular access site. The goal of the preoperative evaluation is to identify potential risks, optimize the patient’s health for surgery, and personalize the surgical plan to ensure a safe and successful outcome. The risk of postoperative complications, including myocardial infarction and arrhythmia, mirror the level of invasiveness of the surgical intervention. Ideally, a multidisciplinary team approach prior to and following major vascular surgery should be considered to optimize patient outcomes [16], 18].

The preoperative assessment clinic provides the ideal environment for informed patient discussion of risk prior to major surgery and shared decision making, including presentation and consideration of all options with the patient. This can be achieved in a timely manner prior to the planned surgical date to allow appropriate multidisciplinary input [19].

Radiographic evaluation plays a vital role in diagnosing complex fractures and the vascular status of the affected extremity and helps guided treatment planning, and monitoring healing. Several imaging modalities are commonly used during assessment [20]. Computed tomography offers superior visualization of comminuted fractures and intra-articular involvement, aiding surgical planning. Three-dimensional reconstruction can further enhance preoperative planning in complex periarticular fractures and evaluate the vascular supply [21]. Magnetic resonance imaging is useful in evaluating associated ligamentous, soft tissue injuries and the vascular supply. MRI and CT are considered largely equivalent in terms of the quality of imaging the vascular situation in the literature. Due to the differing availabilities at different institutions, there is often a preferred choice or standardized procedure for vascular diagnostics in general and to assess possible free flap recipient vessels in particular. However, all techniques largely serve their purpose [22].

Doppler ultrasonography can serve as an effective initial screening tool for vascular compromised extremities. Duplex sonography can provide even more valuable information on flow rates and, in the hands of an experienced practitioner, can be a valuable complement to cross-sectional diagnostics, especially for evaluation of venous conditions [22]. Digital subtraction angiography (DSA) is indicated when vascular compromise is suspected, allowing simultaneous intervention in cases of vascular injury [20] or at later stages to improve vascular inflow to the affected extremity in case of peripheral artery disease. In patients, where metal implants limit the interpretability of cross-sectional imaging, DSA can provide reliable imaging and helps planning the interdisciplinary treatment [22], 23].

### Reconstruction of bone defects – from bone substitutes to autologous bone grafts

The management of bone defects in regenerative medicine and orthoplastic surgery has been the subject of extensive research efforts. Alloplastic, allo- and xenogeneic bone substitutes are alternatives to traditional autologous bone grafts, offering various advantages in bone reconstruction. Autologous bone grafts, while still considered the gold standard for many procedures, have limitations, including donor site morbidity, limited availability, and increased operation time [24]. Bone graft substitutes aim to overcome these drawbacks by providing a scaffold for bone regeneration and/or stimulating bone growth. Natural grafts – comprising autologous, allogeneic, and xenogeneic materials – offer biological advantages, while synthetic alternatives, including biodegradable and non-biodegradable biomaterials, provide structural versatility and reduced immunogenicity [25], 26].

There are different types of bone substitutes: de-mineralized bone matrices are derived from native bone and provide a scaffold but lack the osteogenic and osteoinductive properties of autograft. Synthetic materials including calcium phosphate cements, hydroxyapatite, and bioactive glass offer a scaffold for bone growth and should have osteoconductive, osteoinductive and osteogenic properties. Bio-engineered materials like synthetic scaffolds combined with growth factors or cells to enhance bone regeneration are under investigation [27]. Synthetic bone grafts show promise in achieving comparable outcomes to autografts in radiological, clinical, and quality-of-life measures, while also minimizing complications. Procedures requiring less structural support, such as those involving cancellous bone grafts, appear to benefit most from synthetic options [28].

### Fracture and bone defect stabilization

The choice of adequate osteosynthesis technique in the context of osteoplastic surgery should be performed according to AO principles. The specific technique used depends on several aspects: length of bone defect, choice of bony reconstruction procedure, extension of soft tissue defect, presence of bony and/or soft tissue infection, and general condition of the patient (systemic or local factors that affect immune surveillance, metabolism, and local vascularity) [29].

For open fractures with exposed but well-vascularized bone without significant bone defects and infection, internal osteosynthesis is appropriate. Soft tissue coverage should be performed immediately according to plastic surgery principles to avoid exposed osteosynthesis material or covering of it by vacuum dressings [30].

In the case of longer bone defects reconstruction can be performed using callus distraction according to Ilizarov [31], the membrane technique according to Masquelet [32] (“double barrel”) free vascularized fibular bone graft [33], and 3D-printed scaffolds [34].

In the context of reconstruction with fibula grafts elastic implants must be prioritized, as rigid implants prevent the desired hypertrophy [35]. When using the Ilizarov technique, whether for bone segment transport, limb lengthening or the bifocal compression-distraction technique, external (unilateral or circular) fixators, special nails, or lengthening methods via a nail or plate can be used [36], [37], [38].

Furthermore, external fixation devices stabilize the fracture and avoid large internal metal implants in the situation of infected bone and wound [39]. While external fixation is still justified for severe acute infections, coated implants or calcium sulphate mixed with antibiotics and applied to the implant are increasingly used and may once make temporary external fixation superfluous [40]. In our institution the use of an external fixation device as a definitive osteosynthesis technique is restricted to patients with persisting (despite radical surgical debridement) infections caused by highly resistant and/or biofilm-forming species that are difficult to treat with antimicrobial therapy.

In any case, the aim of osteosynthesis is to provide sufficient stability, which is necessary both for fracture healing and for the prevention and treatment of fracture-related infections. The choice of osteosynthesis technique must consider the need to maintain unrestricted bone perfusion in accordance with the Diamond concept [41], 42].

### Treatment of fracture-associated infections and vacuum therapy until definitive fracture stabilization and defect coverage

Successful treatment of fracture-associated infections depends on appropriate debridement and obliteration of dead spaces with a flap. Particularly in the lower leg, debridement of infected bone and soft tissue should be as radical as necessary, without fear of complex bony and soft tissue reconstruction then being necessary [43].

Topical negative pressure (TNP) therapy has proven to be a useful wound conditioning tool in the treatment of complex extremity injuries. A meta-analysis recently demonstrated the superiority of TNP therapy with intermittent irrigation treatment over conventional TNP therapy in orthoplastic wound management. The more efficient reduction of biofilm probably leads to lower complication rates and higher rates of primary wound closure [44]. The paradigm of mandatory (very) early flap transplantation changed to more differentiated approach, but the controversy over timing of “fix” and in particular “flap” procedures still goes on. In cases with purulent infection, clearance of bacterial load can be achieved through TNP, possibly even more effectively applied in combination with automated instillation of topical wound solutions. Also, tissue demarcation is a prerequisite for rational debridement. However, in patients presenting early with wound/fracture contamination only and no signs of ongoing infection, an early bone cover and in particular soft tissue reconstruction over exposed fractures seem to be beneficial in terms of fracture healing as well as time to rehabilitation. This comes in particular into play, when optimal bone healing can be achieved upon switching the external to an internal fixation (nail, plate), which will then make early/immediate flap cover of those implants mandatory to prevent foreign body infection, biofilm development etc. Despite all its advantages, vacuum therapy should therefore only be used for as long as necessary until the defect is definitively covered in order to reduce the risk of persistent deep infection [45], which in turn underlines the need for early multidisciplinary collaboration.

### Soft tissue reconstruction

After bony stabilization, the soft tissue reconstruction is carried out according to a modular principle and can include different tissue components to cover the defect. Functional restoration often requires a combination of different reconstructive procedures, from alloplastic, allogeneic or xenogeneic tissue replacement materials to free flaps and vascularized bone transplants.

Debridement following severe trauma to the lower extremity of avascular or contaminated/infected bone fragments should be performed expeditiously. All resulting bone defects should ultimately be filled with resorbable ceramic bone substitutes with the addition of antibiotics, allografts or autografts. Autografts include non-vascularized or vascularized bone grafts, the latter being harvested with its vascular pedicle and anastomosed to the recipient artery/vein. This can be done in the form of chimeric, often bipedicular, free flaps, i.e. with simultaneous vascularized bone and soft tissue flaps. Alternatively, two simultaneous individual flaps for bone and soft tissue reconstruction are planned, which are applied one after the other to the same recipient vessel or with the second flap being hooked up to the vascular pedicle of the first flap via an “in-flap”-anastomoses. Combinations of antibiotic-impregnated grafts and non-vascularized corticocancellous bone grafts may be used when the bone defect is only short in extent [46].

If possible, long-distance nerve defects should also be covered at the time of soft tissue defect coverage, most commonly using established autografts (i.e. sural nerve grafts). If postponed secondary flap elevation becomes necessary, which, especially with muscle flaps, often is difficult and fraught with complications. Also, early nerve grafting is in line with the aim of early reinnervation to regain muscular function as early as possible.

When making a therapeutic decision for bone and soft tissue reconstruction, it is important to replace “like with like” and to select the step of the reconstructive ladder with the highest probability of success for the individual patient after weighing up the risk. In addition to complex free flaps, in rare cases this can also be a locoregional flap or a dermis replacement and skin transplant, but in most cases is not possible due to the often extensive compromised skin soft tissue damage and significant area of exposed bone. In addition to mastering the entire range of plastic and microsurgical reconstruction, the management of intercollegiate collaboration is crucial for the success of the reconstruction [3].

### Beyond plastic surgical defect coverage – functional reconstruction with neurotized muscle flaps and reconstruction of peripheral nerve injuries and defects

Traumatic injuries to peripheral nerves are observed in 2–4 % of cases of polytrauma and are a central challenge for the function of an extremity [47]. Particularly in cases of complex injuries to the brachial plexus or several affected nerves, nerve transfers are not always sufficient to achieve the functional reconstruction of an extremity or are not possible due to a lack of donor nerves. In addition, the reconstruction of nerves, whether by direct sutures, nerve grafts or nerve transfers, is only possible within a limited period of up to 12 months after the trauma [48]. At a later point in time, the muscle as the target is atrophied and a reinnervation is not expedient [49]. In such cases, free functional muscle transfer (FFMT) is a valuable addition to the plastic reconstructive surgeon’s repertoire [50].

The transplantation of a free functional muscle involves the reinnervation of the corresponding nerve. Typically, transplantation of the gracilis muscle, supplied by the obturoator nerve, or the latissimus dorsi muscle, with the thoracodorsal nerve, is performed as FFMT. Local nerves or more distant healthy and uninjured nerves are used as a motor donor [51]. More distant nerves are extended to the site of the transplantation via nerve graft, using the sural or saphenous nerve. These procedures are carried out several times, so that the interposition graft is first coaptated to the donor nerve and then, after the expected regeneration of the nerve, the nerve is transplanted [52]. The functional results also vary greatly with the required intensive rehabilitation. In up to 75 % of cases, a strength level of at least MRC 3 can be achieved, in up to 49 % a strength level of MRC 4 [53]. One free functional muscle can usually replace one function. For example, the biceps brachii muscle can be replaced by an FFMT or the flexion of the fingers, although it is possible to isolate the thumb’s flexion by the gracilis muscle [54]. In those cases an arthrodesis of the wrist is mandatory [55].

### Individualized orthoplastic therapy in patients with lower limb peripheral artery disease

Individualized interdisciplinary therapy concepts in the orthoplastic extremity preservation center with integrated rehabilitation strategies and aftercare concepts can help reduce the effects of socioeconomic differences and ensure the reconstruction of the lower extremities at a high level [56].

Compartment syndrome, nerve and vascular injuries are signs of the severity of the trauma, have a negative impact on reconstructive results and may represent an increased risk of thrombosis after microvascular free tissue transplantation [46]. In advanced vascular diseases with compromised macrovascular and microvascular perfusion, restoration of limb perfusion as well as restoration of soft tissue coverage is often necessary in skin-soft tissue defects. The qualitatively and quantitatively poor connection vessel situation with changes in the hemodynamic situation with pronounced arteriosclerosis can lead to flap loss and endangerment of the reconstructive goal if insufficient preoperative evaluation, so that the early involvement of vascular surgery and interventional radiology for the interdisciplinary coordination of preparatory perfusion-improving interventions and/or a combined vascular surgery/plastic surgery approach (e.g. in the form of bypass and flap connection on these) is essential.

Microsurgical management of free flap loss is challenging and requires critical reassessment of risk factors and alternative strategies. This should prompt a rethink of flap choice with a bias toward traditional and robust flaps, the low-threshold use of other recipient vessels including arteriovenous (AV) loops or bypasses, and backup procedures such as vacuum wound therapy or dermal replacement procedures with skin grafting in patients with lower functional needs and the critically ill patients [57]. A constant assessment of the limb function to be achieved, quality of life, hospitalization time with limb preservation vs. possibly reduced morbidity Ablative procedures with modern exoprosthetics are seen as part of the overall concept [58].

## Results

### Exemplary cases of interdisciplinary orthoplastic extremity reconstruction

In the following we will illustrate individualized therapy concepts of orthoplastic interdisciplinary microvascular extremity reconstruction with exemplary cases.

**Figure 1:**
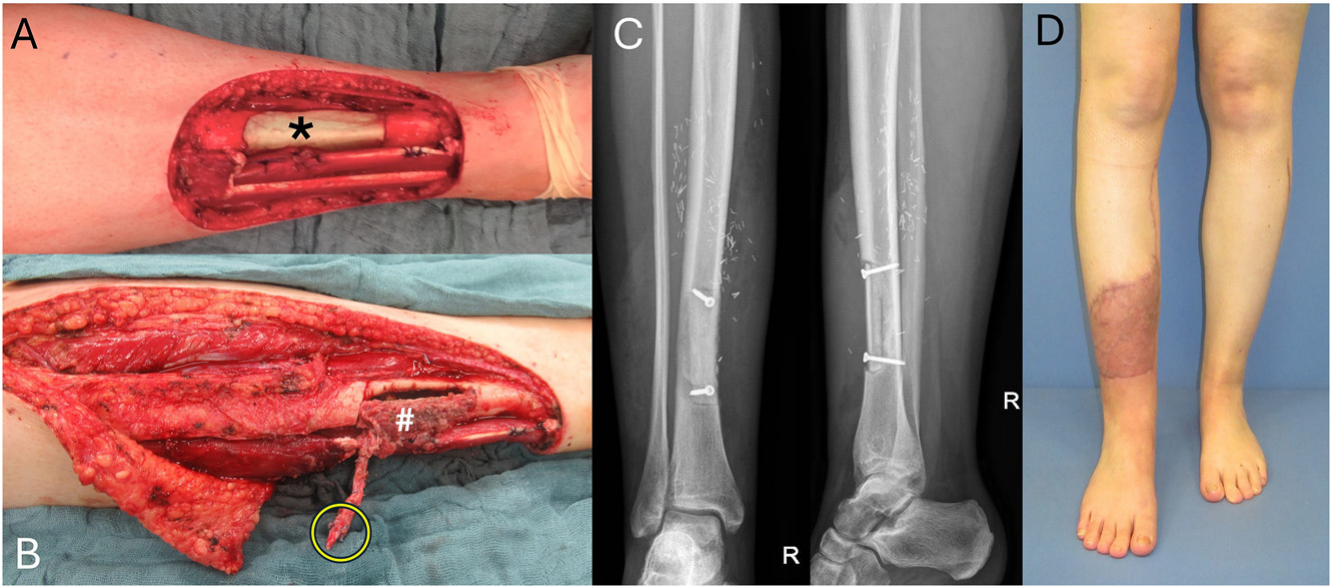
33-Year-old patient presenting with monophasic synovial sarcoma (G2) of the anterior left lower leg. (A) Combined soft tissue – and skin-defect (15x15cm) with tibial bone defect after interdisciplinary complete tumor resection incl. anterior circumferential half of distal tibia + antibiotic-laden cement spacer block implantation (asterisk). (B) Defect reconstruction 1 week later after histopathologically confirmed tumor-free resection margins with free microvascular contralateral fibula (#) for anterior half of tibia (yellow circle=vascular pedicle; press-fit and fixed with two additional compression screws) and free split latissimus dorsi flap transplantation (thoracodorsal pedicle anastomosed anterior tibial artery end-to-side, in-flap anastomosis of fibular pedicle to circumflex scapular branch). (C) X-ray 6 weeks post-operative. (D) Full weight-bearing 10 weeks post-operatively.

Patient 1 is a 33-year-old female presenting with monophasic synovial sarcoma (G2) of the anterior left lower leg. After interdisciplinary complete tumor resection incl. anterior circumferential half of distal tibia + antibiotic-laden cement spacer block implantation a combined soft tissue – and skin-defect with tibial bone defect resulted. Defect reconstruction was performed as an interdisciplinary approach 1 week later after histopathologically confirmed tumor-free resection margins with free microvascular contralateral fibula for anterior half of tibia and free split latissimus dorsi flap transplantation. Full weight-bearing was achieved 10 weeks post-operatively (Figure 1).

**Figure 2:**
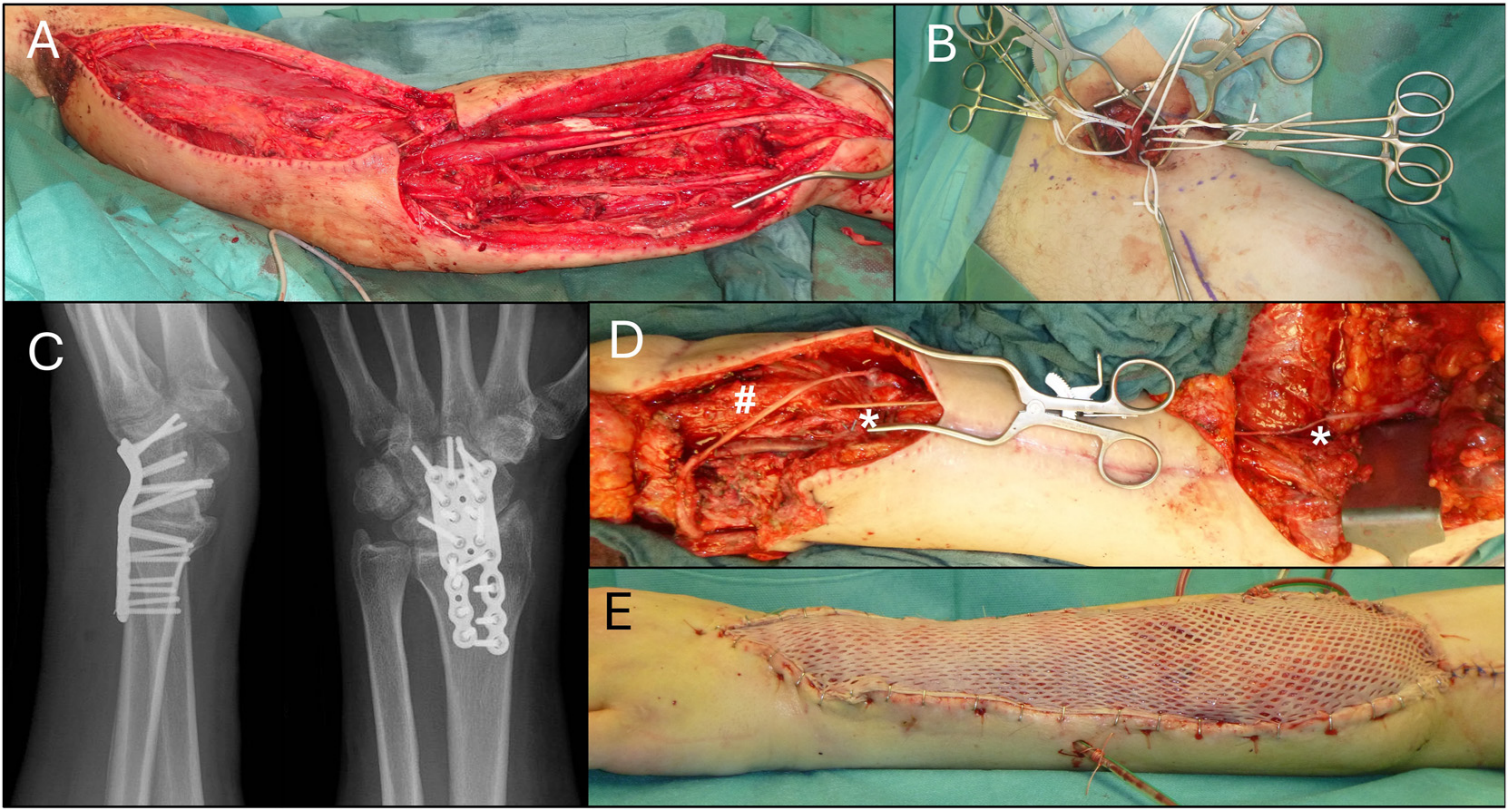
23-Year-old patient with initial treatment for compartment/reperfusion-syndrome of the left lower arm after following plating osteosynthesis of the humeral fracture, reconstruction of brachial artery and vein in a secondary care hospital. (A) Interdisciplinary debridement with neurolysis of ulnar, median and radial nerve, showing complete loss of function at lower arm level due to severe nerve damages and application of topical negative pressure therapy. (B) Supra- and infraclavicular exploration of the brachial plexus revealing neuroma formation of the injured lateral fascicle: intraplexual reconstruction with sural nerve grafts, as well as from there to musculocutaneous and median nerve. (C) Wrist fusion in extension (locked compression plate). (D) 30 cm-sural nerve autograft from central nerve stump of thoracodorsal nerve (previously innervating the harvested latissimus dorsi muscle) at upper arm level (*) down to volar lower arm (*) as a preparation for later free functional gracilis transfer for finger flexion (#=sural nerve graft between motor branch to latissimus dorsi muscle to proximal ulnar nerve as donor). (E) Transplantation of free functional latissimus dorsi muscle to restore finger extension (neurotization via sural nerve graft to ulnar nerve) covered with split thickness skin grafts.

Patient no. 2 is a 23-year-old patient who had ORIF of a left humeral shaft fracture with simultaneous reconstruction of brachial artery and vein in a secondary care hospital. After initial treatment for compartment/reperfusion-syndrome of the left lower arm he presented with an extremity like “Volkmann ischemic contracture”. An interdisciplinary debridement with neurolysis of ulnar, median and radial nerve verified complete loss of function at lower arm level due to severe proximal nerve damage in the infraclavicular plexus level, as well as structural loss of the median and radial nerve reaching down to the forearm. TNP therapy system was applied. In the following operations we performed supra- and infraclavicular exploration of the brachial plexus revealing neuroma formation of the injured lateral fascicle and proceeded with an intraplexual reconstruction with sural nerve grafts, as well as grafting from there to musculocutaneous and median nerve. To stabilize the wrist, a wrist fusion in extension with locked compression plate followed. Furthermore, a 30 cm-sural nerve autograft from the central nerve stump of the thoracodorsal nerve was implanted as preparation for later free functional gracilis transfer for finger flexion. In the same operation we transplanted a free functional latissimus dorsi muscle to restore finger extension with neurotization via a sural nerve graft to the proximal ulnar nerve, covered with split thickness skin grafts. The latissimus flap was chosen for extensor reconstruction since a large soft tissue defect on the dorsal forearm (A, E) could be addressed at the same time, while the (smaller) gracilis muscle was spared for reanimation of the finger flexion on the volar aspect of the forearm, where no soft defect was present (Figure 2).

**Figure 3:**
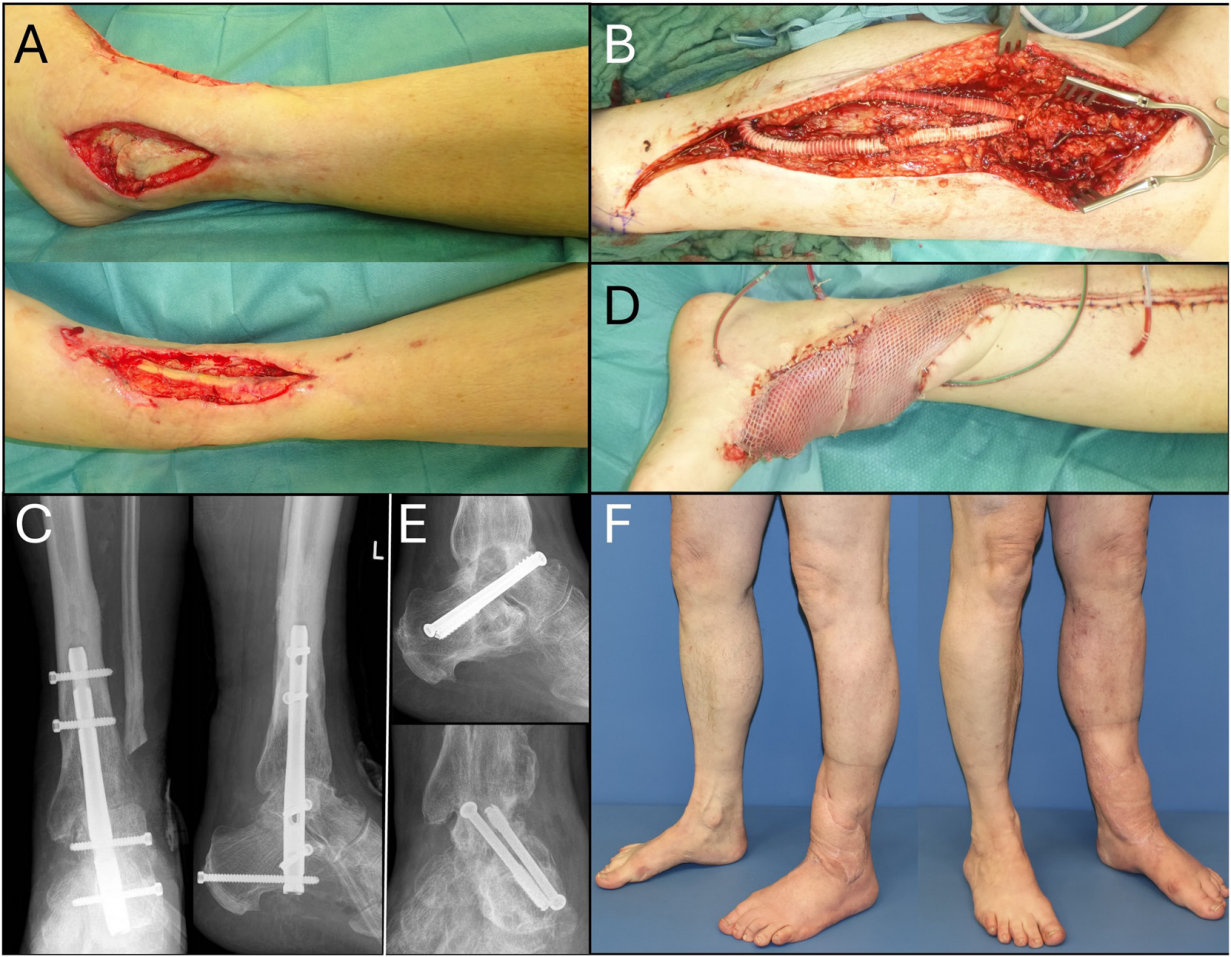
65-Year-old patient upon debridement presenting with chronic osteomyelitis 30 years after bimalleolar open ankle fractures. (A) Upon first interdisciplinary debridement and initiation of topical negative pressure therapy. (B) Due to occlusion of the left AFS, proximal thromboendarterectomy and patch plasty of the common femoral artery as well as femoropopliteal bypass was performed first by the Department of Vascular Surgery, followed by creation of an arteriovenous-loop from the popliteal level using a greater saphenous vein autograft. The AV-loop was pulled through a vascular PTFE-allograft for protective reasons. (C) After 7 days a tibiotalocalcaneal arthrodesis using an intramedullary nail fixation was done. (D) Finally soft tissue reconstruction by free latissimus dorsi muscle flap transplantation with anastomosis to the apex of the loop in end-to-end fashion was performed. (E) After 1 year nail was removed and replaced by 2 partially threaded screws due to suspicion of talocalcaneal pseudarthrosis. (F) 2 years after orthoplastic reconstruction the patient is ambulatory without crutches an no clinical sign of infect recurrence.

Patient 3 demonstrates extremity reconstruction in a 65-year-old male patient with peripheral artery disease. He presented with chronic osteomyelitis 30 years after bimalleolar open ankle fractures. After initial interdisciplinary debridement we commenced topical negative pressure (TNP) therapy. Due to occlusion of the left superficial femoral artery (AFS), proximal thromboendarterectomy and patch plasty of the common femoral artery as well as femoropopliteal bypass was performed first by the Department of Vascular Surgery, followed by creation of an arteriovenous-loop (AV-loop) from the popliteal level using a greater saphenous vein autograft. The AV-loop was pulled through a vascular PTFE-allograft for protective reasons, since from our experience this outweighs the additional risk for foreign body infection, as well as impeded exposure of the AV-loop in case of revision, in selected cases. After 7 days a tibiotalocalcaneal arthrodesis using an intramedullary nail fixation was done. Finally soft tissue reconstruction by free latissimus dorsi muscle flap transplantation with anastomosis to the apex of the loop in end-to-end fashion was performed. After 1 year, nail was removed and replaced by 2 partially threaded screws due to suspicion of talocalcaneal pseudarthrosis 2 years after orthoplastic reconstruction the patient is ambulatory without crutches an no clinical sign of infect recurrence. Transit time as well as pre- and post-flap AV-loop flow characteristics are assessed by using an intra-operative flow probe, with changes in flow dynamics being under investigation in a separate study (Figure 3).

### Proposed algorithm for individualized therapy concepts in the orthoplastic extremity reconstruction

**Figure 4:**
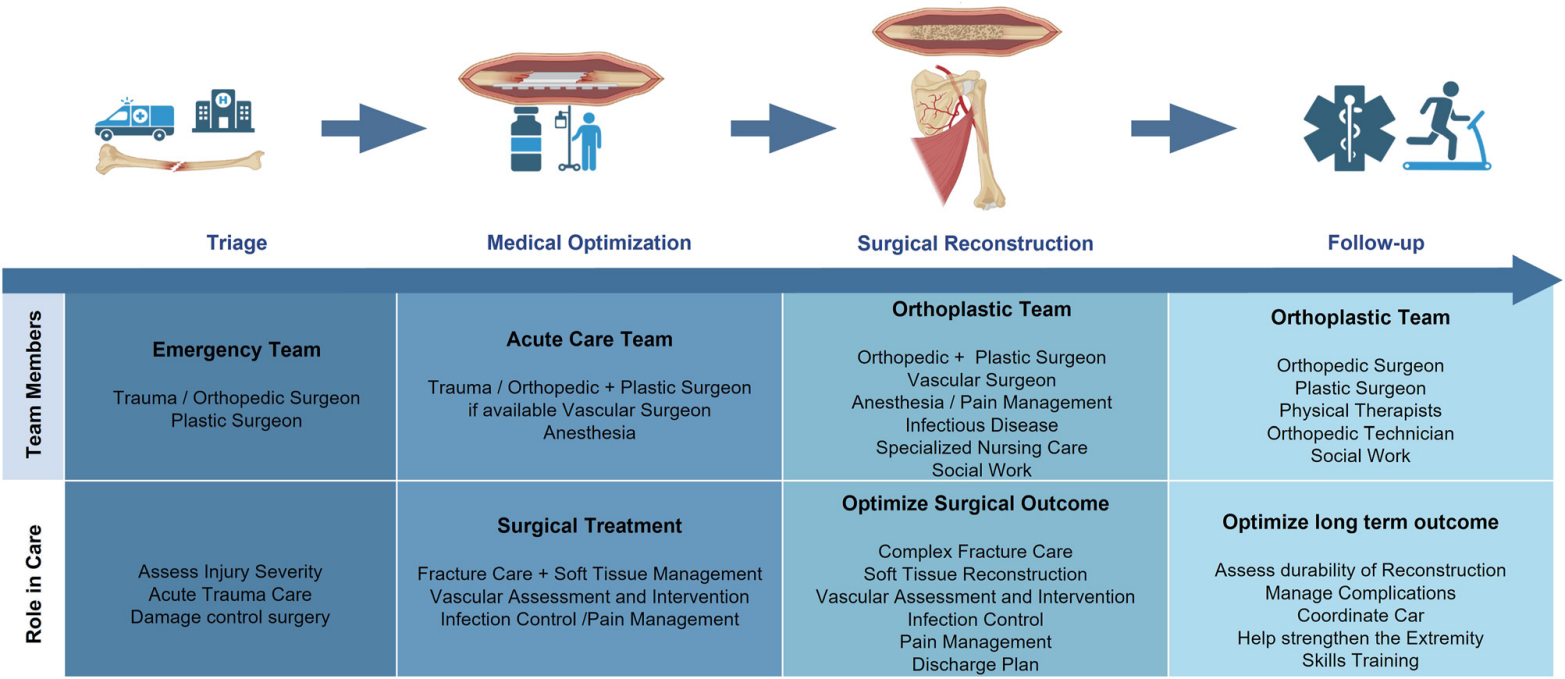
Proposed algorithm for individualized therapy concepts in the orthoplastic extremity reconstruction; modified according to Refs. [5], 56].

The goal of limb salvage is to have the patient achieve the best possible function that allows daily activities (e.g. walking/running) and the ability to perform tasks that require strength and dexterity. This is a complex process requiring the work of a multidisciplinary team practicing the principles of good wound care, orthoplastic thinking, and strategizing on future function [5], 56]. Decision-making strategy for lower extremity limb salvage according to functionality and other considerations and strategy for functional limb salvage – the “orthoplastic thinking” is provided in Figure 4.

## Conclusions

The treatment of traumatic injuries of the lower extremities requires a range of skills from orthopedic/trauma surgery and plastic surgery, as well as, where appropriate, vascular surgery, to optimize the restoration of form and function. The orthoplastic approach shortens the time to definitive osteosynthesis, reduces the risk of wound infection and osteomyelitis, and increases the likelihood of limb salvage compared to the non-orthoplastic approach. Orthoplastic treatment of traumatic injuries of the lower extremities offers a synergistic model for optimizing and accelerating definitive skeletal fixation and flap-based soft tissue coverage to restore the form and function of the extremities [58]. This simultaneous, or in some steps sequential, orthoplastic approach and the associated need for early coordination of a joint interdisciplinary treatment concept will be concretely demonstrated in the lecture using exemplary cases of extremity reconstruction.
